# Population transcriptomic analysis identifies the comprehensive lncRNAs landscape of spike in wheat (*Triticum aestivum* L.)

**DOI:** 10.1186/s12870-022-03828-x

**Published:** 2022-09-21

**Authors:** Guang Yang, Pingchuan Deng, Qifan Guo, Tingrui Shi, Wenqiu Pan, Licao Cui, Xiaoqin Liu, Xiaojun Nie

**Affiliations:** 1 State Key Laboratory of Crop Stress Biology in Arid Areas, College of Agronomy and Yangling Branch of China Wheat Improvement Center, Northwest A&F University, Yangling, 712100 Shaanxi China; 2 College of Bioscience and Engineering, Jiangxi Agricultural University, Nanchang, 330045 Jiangxi China; 3 Peking University Institute of Advanced Agricultural Sciences, Weifang, 261325 Shandong China

**Keywords:** Wheat, Spike, Population transcriptome, lncRNA, GWAS

## Abstract

**Background:**

Long noncoding RNAs (lncRNAs) are emerging as the important regulators involving in growth and development as well as stress response in plants. However, current lncRNA studies were mainly performed at the individual level and the significance of it is not well understood in wheat.

**Results:**

In this study, the lncRNA landscape of wheat spike was characterized through analysing a total of 186 spike RNA-seq datasets from 93 wheat genotypes. A total of 35,913 lncRNAs as well as 1,619 lncRNA-mRNA pairs comprised of 443 lncRNAs and 464 mRNAs were obtained. Compared to coding genes, these lncRNAs displayed rather low conservation among wheat and other gramineous species. Based on re-sequencing data, the genetic variations of these lncRNA were investigated and obvious genetic bottleneck were found on them during wheat domestication process. Furthermore, 122 lncRNAs were found to act as ceRNA to regulate endogenous competition. Finally, association and co-localization analysis of the candidate lncRNA-mRNA pairs identified 170 lncRNAs and 167 target mRNAs significantly associated with spike-related traits, including lncRNA.127690.1/TraesCS2A02G518500.1 (*PMEI*) and lncRNA.104854.1/TraesCS6A02G050300.1 (*ATG5*) associated with heading date and spike length, respectively.

**Conclusions:**

This study reported the lncRNA landscape of wheat spike through the population transcriptome analysis, which not only contribute to better understand the wheat evolution from the perspective of lncRNA, but also lay the foundation for revealing roles of lncRNA playing in spike development.

**Supplementary Information:**

The online version contains supplementary material available at 10.1186/s12870-022-03828-x.

## Background

It has been widely demonstrated that the eukaryotic genome could transcribe a huge number of non-coding RNAs (ncRNAs), which have the indispensable regulatory functions on the epigenetic control of developmental trajectories [1, 2]. ncRNAs can be classified into small RNAs, medium-sized ncRNA and long ncRNA based on their sizes [3]. Long ncRNA (lncRNA) is defined as the transcripts with the length of longer than 200 bp and having no discernible coding potential [4], which can be further classified as long intergenic ncRNAs (lincRNAs), intronic ncRNAs (incRNAs), sense-lncRNAs and natural antisense transcripts (NATs) transcribed from the complementary DNA strand of the associated genes [4, 5]. LncRNAs were long considered little beyond transcriptional noise; however, there is increasing evidence that lncRNAs played the key roles in diverse biological processes across eukaryotes [4, 6]. Generally, lncRNA was reported to function as either *cis-* or *trans-* element through affecting neighboring loci or performing distal regulatory effect, as enhancer, decoy, guide or scaffold for protein complexes to control gene expression [3]. With the development of next generation sequencing technologies, extensive studies have conducted in genome-wide characterization and functional validation of lncRNAs. In plants, lncRNA has been functionally characterized as participating in flowering, sexual reproduction, root organogenesis, responding to biotic and abiotic stresses and so on [7, 8]. For instance, 177 differentially expressed lncRNAs were identified to be related to Ca2 + channel blocking based on RNA-seq data of wheat seeds [9]. Forty-eight *cis-*lncRNA and 31 *trans-*lncRNA were found to control leaf-color of *Ginkgo biloba* L [10]. Additionally, five differentially expressed lncRNAs that related to wheat salt tolerance were also verified by using the lncRNA sequences and virus-induced gene silencing methods, of which two lncRNAs knockdown caused the plants to exhibit sensitivity to alkaline stress, and three knockdown increased the salt tolerance [11]. Although many studies on lncRNA identification have been reported in plants, the biogenesis and function of plant lncRNAs, especially those underlying important agricultural traits, is not well understood compared to human and other model animals. Furthermore, most of the plant lncRNAs were characterized in the individual genotype level rather than at the population transcriptome level, limiting the better understanding their comprehensiveness and evolution.

Wheat is one of the most important staple crops worldwide, accounting for approximately 30% of the global cultivated area, and providing 20% of the world's food consumption [12]. It is estimated that wheat production needs to be increased by 1.5% annually to meet the demands of 9 billion people in 2050 [13]. Consequently, improvement of wheat’s yield is urgently needed. Similar to other cereal crops, such as maize, rice and barley, the yield of wheat is a complex trait determined by spike number per plant, grain number per spike (GN) and one-thousand kernel weight (TKW), all of which are determined largely by spike architecture and are thus considered as spike-related traits. In light of their importance, extensive studies have been performed to identify the genes and loci underlying spike architecture and spike-related traits. Based on the 90 K iSelect SNP genotyping assay, 306 loci were found to be significantly associated with heading and flowering dates in 375 Chinese common wheat and these loci were also associated with spike length, peduncle length, fertile spikelet number, cold resistance, and tiller number [14]. The *Q* locus, encoding an AP2-like transcription factor, is one of widely studied domestication genes regulating spike compactness, brittle rachises and grain morphology [15]. *GW2*, encoding an E3 RING ligase, plays an important role in regulating grain number and size in wheat and other cereal crops [16].

Some lncRNAs were also found to involve in regulating spike development and yield-related traits. In Arabidopsis, a long intron noncoding RNA functioned as the crucial regulator in mediating epigenetic inhibition of *FLC* by vernalization to control flowering [17, 18]. Furthermore, one lncRNA in rice, *XLOC_057324*, is demonstrated to play a role in panicle development and fertility [19]. Two hundred and thirty-seven and 20 lncRNAs were identified to related with pollen mother cell (PMC) and embryo sac mother cell (EMC) meiosis in autotetraploid rice [20]. In wheat, 8,889 expressed lncRNAs, including 2,753 differentially expressed ones, were identified during wheat spike development at six stages in the elite variety Zhengmai 366, showing their important roles in wheat spike development [21]. However, it was conducted only in one wheat genotype, and the genetic variations and evolutionary characteristics of wheat lncRNA is still elusive.

Here, we used the 186 strand-specific RNA-seq data from 93 wheat genotypes to identify the lncRNAs in the spike at heading and flowering stage. The genomic organization, genetic variations and evolution features as well as regulatory network of these wheat lncRNAs were systematically characterized. Furthermore, we integrated GWAS and known QTLs to identify the candidate lncRNAs associating with spike-related traits. It is the first study to characterize lncRNA at the population transcriptome level, which provided a comprehensive lncRNA landscape of wheat spike, and also facilitate to reveal the function of lncRNA on yield-related traits in wheat and beyond.

## Results

### Genome-wide identification of lncRNAs in wheat spike

**Fig. 1:**
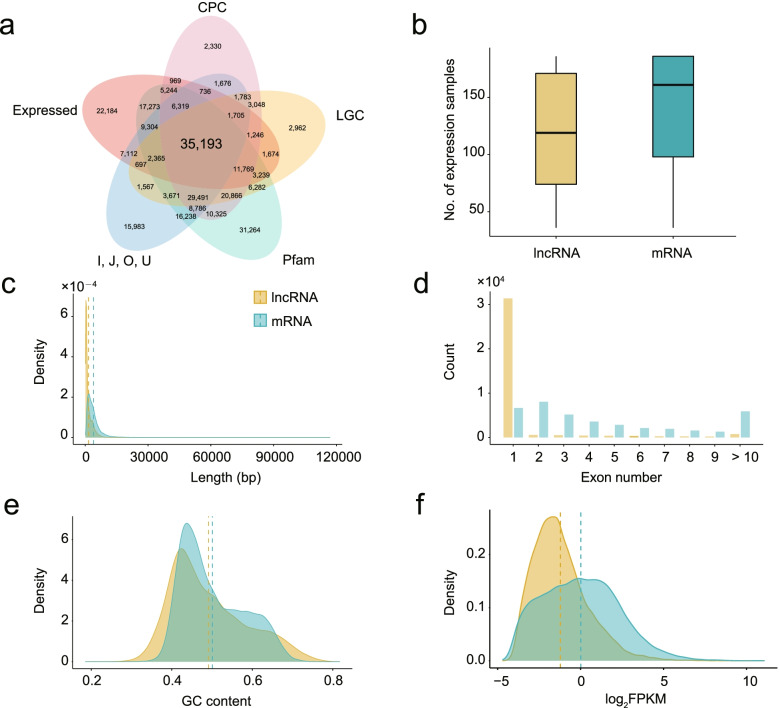
Comparison of properties of lncRNA and protein-coding genes. **a** Identification of lncRNA in wheat according to CPC tool, LGC tool as well as the Protein Families database (Pfam); **b** The number of lncRNA expressed and mRNA expressed samples; **c** Transcript size of lncRNA and mRNA; **d** The number of exons in lncRNA and mRNA transcripts; **e** GC content of lncRNA and mRNA transcripts; **f** Comparison of log_2_(FPKM) values of lncRNA and mRNA

To comprehensively identify lncRNA in wheat spike, we used a total of 186 RNA-seq datasets of the spike samples collected from 93 genotypes to identify the lncRNA. Totally, 4.6 Tb clean reads were obtained to align to the wheat reference sequence (IWGSC Refseq v1.1) and all samples had the average alignment rate of more than 90%. Reference guided transcriptome assembly strategies were further used to identify lncRNAs, resulting in 35,913 lncRNAs obtained (Fig. 1a, Additional file 1: Table S1). Among them, 4,146 (11.5%) lncRNAs were found in the plant lncRNA database (http://greenc.sequentiabiotech.com/wiki2/Main_Page) and the average sequence identity was 92.4%.

**Fig. 2:**
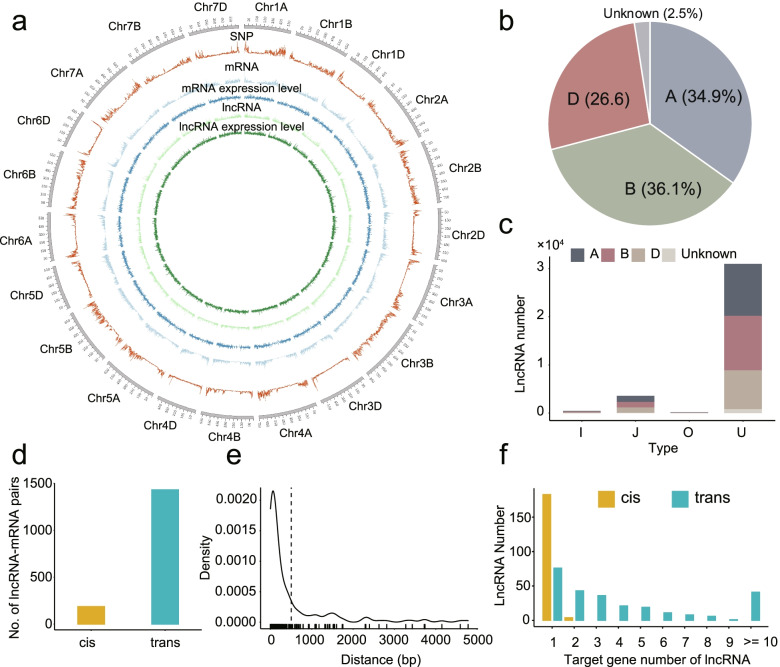
Genomic organization and characteristics of lncRNAs-mRNAs pairs in wheat. **a** Summary of SNP density, mRNA density, mRNA expression level, lncRNA density and lncRNA expression; **b** Subgenome distribution of lncRNAs in wheat A, B and D genomes; **c** Statistic of lncRNA types; **d** The number of *cis-* and *trans-* regulatory lncRNA-mRNA pairs; **e** Distribution of distance between *trans-*lncRNAs and target mRNAs; **f** Distribution of the number of lncRNA target genes

Compared to mRNA, these lncRNAs showed different sequence characteristics. The average expressed samples of lncRNAs was 119, whereas that of mRNA was 161 (Fig. 1b). The median length of lncRNAs and mRNA were 1,584 bp and 3,953 bp, respectively (Fig. 1c). Meanwhile, the exon number of lncRNA was less than that of protein-coding transcripts and the majority of lncRNA transcripts (89.2%) had only one exon (Fig. 1d). However, they have no significant difference on GC content (Fig. 1e). The median expression levels of mRNAs were higher than that of lncRNAs (Fig. 1f). Furthermore, the mRNA and lncRNA density in different chromosome and subgenomes were calculated with 1 Mb interval (Fig. 2a). The results showed that the whole genome had an average of 2.71 mRNAs and 2.41 lncRNAs per Mb, respectively. Meanwhile, the number of mRNA on each chromosome was moderately correlated (*R* = 0.40) with the number of lncRNA through regression analysis. Moreover, most lncRNAs were identified on the B subgenome (36.1%), followed by the A (34.9%) and D subgenome (26.6%) (Fig. 2b). The mRNA density of D genome was significantly higher than that of A and B subgenomes (A: 2.53/Mb; B: 2.48/Mb; D: 3.33/Mb), while the opposite trend was found on lncRNAs that D subgenome displayed the lowest lncRNA density among the three subgenomes (A: 2.49/Mb; B: 2.45/Mb; D: 2.37/Mb). Among all lncRNA, long intergenic noncoding RNA (lincRNA) (30,974) had the largest abundance (Fig. 2c). Interestingly, 36 lincRNA were identified in 15 centromeric intervals, of which 2D centromeric region had the most lincRNAs. Regression analysis showed a significant correlation between lincRNA abundance and centromere region size (*R* = 0.47, *P*-value = 0.0054). The mean expression level (FPKM) of these lincRNA was 1.25, which was relatively lower than that of other lncRNAs.

### Identification of the targets of these lncRNAs

Correlation of expression level between lncRNAs and protein-coding genes can imply their common involvement in some biological processes [22]. Then, a total of 1,619 lncRNA-mRNA pairs (187 *cis*- and 1,432 *trans-*pairs) were identified based on the combination of physical position, expression correlation and LncTar tool analysis, including 443 lncRNA and 464 mRNA (Fig. 2d, Additional file 1: Table S2). The distance between *cis-*lncRNAs and target mRNAs ranged from 1 bp to 4,782 bp with a mean length of 503 bp (Fig. 2e). The *cis*-lncRNAs were found to be one to one targeted on mRNA, while one-to-many was found between *trans*-lncRNAs and their targets (Fig. 2f). Subsequently, GO enrichment analysis showed that these targeting genes significantly enriched into pollen tube development (GO:0048868, 8.90E-16), pollen tube growth (GO:0009860, 1.50E-13), and cell tip growth (GO:0009932, 3.70E-13) (Additional file 1: Table S3, Additional file 2: Figure S1a). KEGG analysis showed that they enriched into many metabolic pathways, including sphingolipid metabolism, nitrogen metabolism, fatty acid metabolism (Additional file 2: Figure S1b).

### Analysis of sequence conservation of lncRNA in wheat and other species

**Fig. 3:**
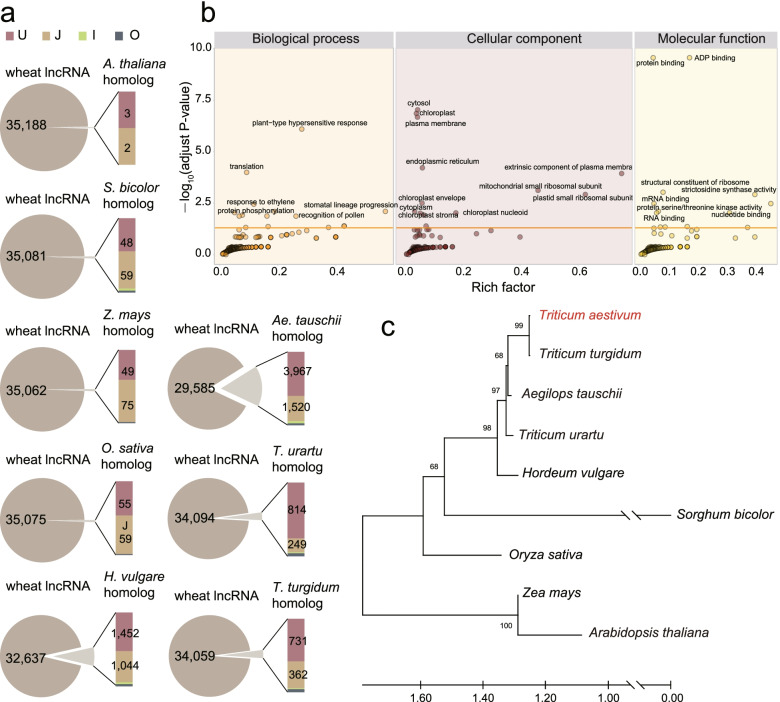
Conservation and evolution of lncRNAs in 9 plant species. **a** Sequence conservation of wheat lncRNAs based on results of homolog alignment; **b** GO enrichment analysis of target mRNA of all homolog lncRNA; **c** Phylogenetic tree was constructed using single copy lncRNA in the 9 species

To understand the evolution of lncRNA, we performed similarity alignment and conservation analysis of the lncRNA among *A. thaliana*, sorghum (*S. bicolor)*, maize *(Z. mays)*, rice *(O. sativa),* barley *(H. vulgare)*, *Ae. tauschii*, *T. urartu*, emmer wheat *(T. turgidum)* and common wheat. Results showed that 8,133 out of 35,193 wheat lncRNAs (23.1%) possessed homologous relationships with other species (Additional file 1: Table S4). The most homologous relationships were identified between *T. aestivum* and *Ae. tauschii*, followed by *H. vulgare*, *T. turgidum, T. urartu, Z. mays, O. sativa*, *S. bicolor* and *A. thaliana*. Most of homologous lncRNA were located in intergenic region (Fig. 3a). GO enrichment analysis of target genes of homologous lncRNAs showed that conserved lncRNAs were involved in important biological process, such as auxin biosynthetic process (GO:0009851), protein phosphorylation (GO:0006468), and recognition of pollen (GO:0048544) (Fig. 3b).

In order to reduce the impact of different methods on the identification of homologous lncRNAs. We further investigated the conservation of these lncRNAs across different species genome. Wheat lncRNAs were aligned to the genome sequence of these eight species, and the conservation score was calculated based on sequence coverage and identity. Results showed that the most conservation was found between *T. turgidum* and *T. aestivum* (Additional file 1: Table S5). Based on the single copy lncRNA among the nine species, a phylogenetic tree was constructed (Fig. 3c). It showed that wheat also displayed the closest phylogenetic relationship with its progenitors emmer wheat, *Ae. tauschii*, *T. urartu,* and followed by barley, which was consistent with the evolutionary history of these species.

### Population genetic analysis of lncRNAs and targeted mRNAs

**Fig. 4:**
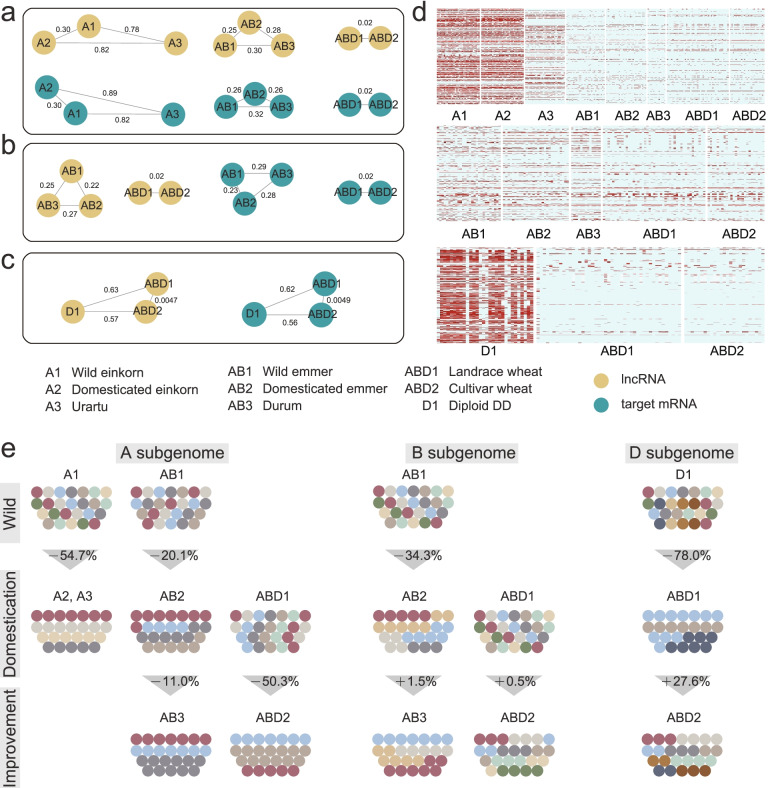
Population divergence and genetic diversity of lncRNAs and target mRNAs across the nine population groups. **a-c** Population divergence between each group in each subgenome. The values between the circles represent fixation index (*F*_st_); **d** The SNP density of lncRNA and target mRNA in each subgenome; **e** Change of genetic diversity of *Triticum* species and *Aegilops*. Plus and minus signs represent the increase and decrease of diversity

Based on genotyping data of 261 accessions from *Triticum* and *Aegilops* genera [23] (Additional file 1: Table S6), the genetic variations of lncRNA-mRNA pairs in wheat and its eight progenitors were investigated (see Methods section for more details). The fixation index (*F*_st_) and nucleotide diversity (π) were calculated (Fig. 4a-c). For each group, the *F*_st_ value between A2 and A3 group (lncRNA: 0.82; mRNA: 0.89) tended to be largest, indicating a larger degree of divergence. The lowest *F*_st_ was observed between ABD1 and ABD2 (lncRNA: 0.0047; mRNA: 0.0049). For each subgenome, the B subgenome had the lowest degree of divergence and there was no significant difference between A and D subgenome. The π of wild species was always larger than that of cultivated species in lncRNA and mRNA targets of different populations and subgenomes. The average π value of B subgenome was larger than that of A and D subgenome (Additional file 1: Table S7). Then, SNP density of lncRNAs and mRNAs showed that genetic bottleneck events were found in A and D subgenome (Fig. 4d). The shifting of genetic diversity also indicated more genetic bottleneck events appeared in domesticated process (Fig. 4e). During domestication and improvement processes, the genetic diversity continued to shrink and the large bottleneck event occurred between A1 and A2/A3. In contrast, the B and D genomes showed the bottleneck event in domestication but genetic expansion in improvement, especially between landraces and varieties. The durum wheat and hexaploid bread wheat appeared to have more genetic diversity after improvement, indicating the breeders would select specific genetic resource according to their needs in different environments.

By phylogenetic analysis using these genetic variations, we found each subgroup, expecting ABD1 and ABD2, had the consistent topology (Additional file 2: Figure S2-S4), indicating that there was potential gene flow between ABD1 and ABD2 group Furthermore, we identified the haplotype organization and frequency of each lncRNA- mRNA pair in these populations based on the resequencing data (Additional file 1: Table S8). A total 192 lncRNA and 213 mRNA were found to have the genetic variations among these populations. Among them, 3 lncRNAs and 8 mRNAs were identified to have diverse haplotypes in different populations. It is showed that haplotype GGGGGGTTGGGGTTCCCCTTGGAACC (76.92%) was the major haplotype in the AB3 population, while GGGGGGTTGGGGTTCCCCTTGGAAAA was the most abundant in ABD1 (93.33%) and ABD2 (100%) populations. These results showed that these lncRNAs suffered a stronger genetic bottleneck and some loci associating with important traits may be added during the processes of domestication and improvement.

### Endogenous competitive role of lncRNA in regulatory network

**Fig. 5:**
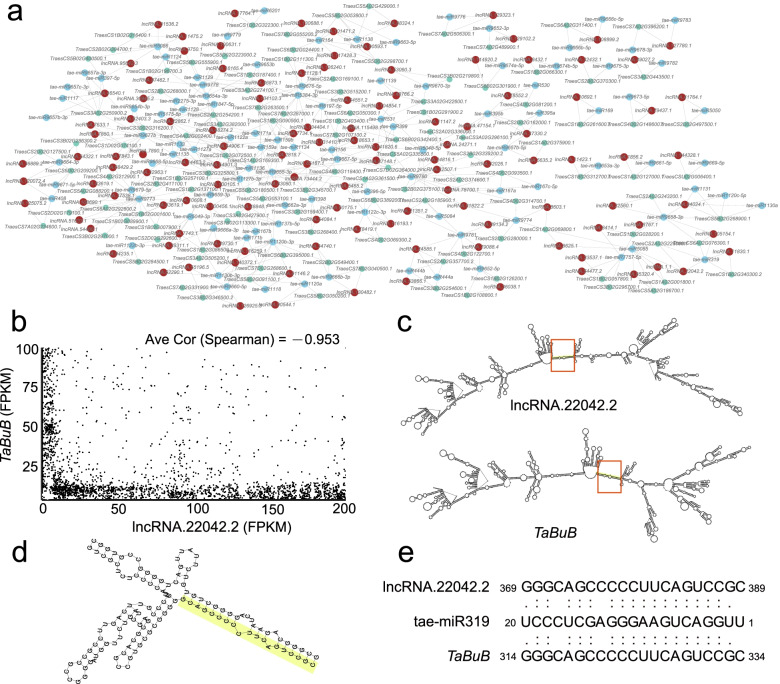
Analysis of target mimicry of lncRNA. **a** Interaction network of lncRNAs, tae-miRNAs and mRNAs (brown, lncRNA; skyblue, miRNA; grey, mRNA); **b** The scatter diagram of lncRNA-mRNA pairs expression level in each sample; **c** The secondary of lncRNA.22042.2 and *TaBuB* (TraesCS2A02G226000.1); **d** miRNA binding region of lncRNA and mRNA; **e** The sequence of lncRNA.22042.2, tae-miR310 and *TaBuB*

It is reported that lncRNA can also function as the competing endogenous RNAs [24, 25]. lncRNAs can act as decoys for miRNAs to competitively inhibit their interaction with target mRNAs. Furthermore, we constructed the ceRNA network of wheat spike-related lncRNA, showing the interaction relationships among lncRNA, miRNA and mRNA in wheat spike (Fig. 5a).

A total of 122 lncRNAs, 102 miRNAs and 119 mRNAs were contained in ceRNA network and 238 unique triangle pairs of lncRNA-miRNA-mRNA were identified (Additional file 1: Table S9). For instance, lncRNA.2204.2 was competitive in tae-miR319-TraesCS2A02G226000.1(*TaBuB*) pairs, showing the significantly negative impact between the expression level of lncRNA.2204.2 and *TaBuB* (Fig. 5b). The 369 bp to 389 bp of lncRNA.2204.2 cDNA is the same as the 314 to 334 bp of *TaBuB*, which can be cleaved by tae-miR319 (Fig. 5c-e). Moreover, tae-miR9773 showed the most interaction pairs in the ceRNA network, which displayed interaction with tae-miR9667b, tae-miR9780, tae-miR159, tae-miR156, tae-miR1127b and tae-miR1120b, indicating its important role in the lncRNA-miRNA-mRNA regulatory network.

### Functional analysis of lncRNA-mRNA pairs

**Fig. 6:**
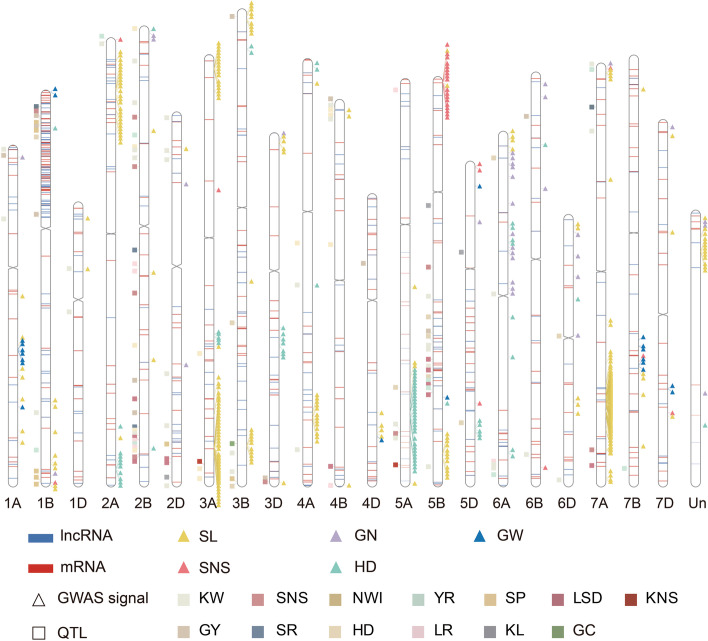
Integration of lncRNAs, target mRNAs, GWAS signals and known QTLs

To obtain some clues about the function of these lncRNA, we combined the co-localization analysis with 124 known QTLs and GWAS analysis of the genetic variations in 1,619 lncRNA-mRNA pairs with 5 agronomic traits to identify the causal lncRNAs underlying yield-related traits. As the results, 54 lncRNAs and 52 mRNAs were found to associate with agronomic traits by GWAS analysis, and formed 318 lncRNA-mRNA pairs (Additional file 1: Table S10). Meanwhile, 130 lncRNAs and 128 mRNAs were found to overlap with known QTLs (Additional file 1: Table S11). Overall, 14 lncRNAs and 13 mRNAs were shared by GWAS signals and known QTL regions. GWAS results showed that most lncRNAs were associated with spike length, followed by heading date (Fig. 6). For example, lncRNA.7517.1 was related to heading date, and its expression level was negatively correlated with that of its target TraesCS5B02G326100.1, which is the ortholog of *AtSCL5,* a transcription factor involving in plant development [26].

Furthermore, we analyzed the genetic introgression among these lncRNAs according to the introgression regions reported by previous study [27]. A total of 85 lncRNAs and 80 mRNAs were found in introgression intervals (Additional file 1: Table S12). Meanwhile, we performed fisher's exact test and indicated a significant correlation (*P*-value = 2.8e-14) between the introgression intervals and lncRNA-mRNA pairs. Interestingly, the distribution of lncRNA-mRNA pairs enriched in chromosome 1BS (Fig. 6). Furthermore, a total of 65 loci (lncRNA, 33; mRNA, 32) on 1B/1R region were divided into two clusters in all experimental samples based on expression abundance (Additional file 2: Figure S5a). The expression level of lncRNA and mRNA between non-1B/1R samples (158) and 1B/1R samples (28) was also significantly differential (Additional file 2: Figure S5b), among them, because the reference genome is a non-1B1R line, the expression level of lncRNAs and mRNAs in the non-1B1R samples were significantly higher. GO enrichment analysis showed that they enriched into chloroplast envelope (GO:0009941), cytosol (GO:0005829), maintenance of DNA methylation (GO:0010216) and chloroplast stroma (GO:0009570) (Additional file 2: Figure S5c). Finally, we performed qRT-PCR analysis of five randomly selected 1B/1R lines-specific lncRNAs and five non-1B/1R lines-specific lncRNAs in ten wheat accessions (five 1B/1R and five non-1B/1R lines). The results showed that five 1B/1R lines-specific lncRNAs were highly expressed in 1B/1R group but low or even not expressed in non-1B/1R lines, while the expression patterns of non-1B/1R lines-specific lncRNAs was opposite (Additional file 2: Figure S6).

### LncRNA-mRNA pair related to heading date

**Fig. 7:**
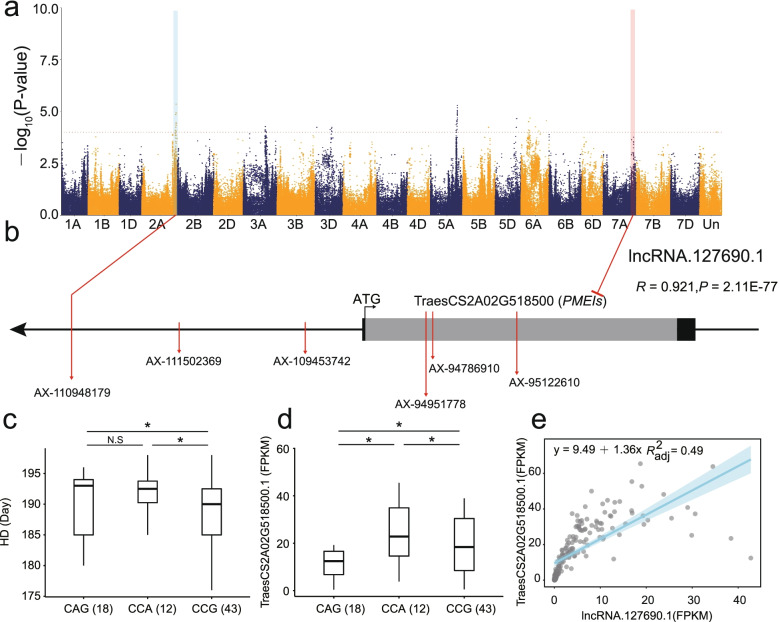
Key lncRNA-mRNA pair related to heading and flowering stage. **a** Manhattan plot of heading and flowering stage; **b** Gene structure and SNP position of TraesCS2A02G518500.1; **c** The phenotype value of heading and flowering stage under each haplotype; **d** The expression level of lncRNA.127690.1 under each haplotype; **e** The regression analysis of expression level between lncRNA.127690.1 and TraesCS2A02G518500.1. (*, *P*-value ≤ 0.05; N.S, not significant)

Through GWAS analysis, a total of 5 lncRNAs and 9 target mRNAs were found to be related to heading date. One GWAS signal that associated with wheat heading date (AX-110948179, C to T, Chr2A:738974737, *P*-value = 8.36E-05) was found in the upstream of TraesCS2A02G518500.1, which was co-expressed with lncRNA.127690.1 (Fig. 7a). We found that three non-synonymous SNP variations were located in the coding regions of this gene (Fig. 7b). Furthermore, three major haplotypes were constructed based on these three SNPs (AX-94951778: C > A, Asp > Glu; AX-94786910: C > A, Asn > Lys; AX-95122610: G > A, Arg > Gln). Interestingly, haplotype CCG corresponded to the shorter heading date, and a higher expression level of TraesCS2A02G518500.1, while CAG genotype had longer heading date and a lower expression level (Fig. 7c and d). The phylogenetic tree based on SNP of TraesCS2A02G518500.1 showed genetic flow might occur between tetraploid wheat and hexaploid wheat populations (Additional file 2: Figure S7). Interestingly, TraesCS2A02G518500.1 could encode a pectin methylesterase inhibitor protein. In rice, *PMEI* has been demonstrated to associate with heading [28]. Moreover, lncRNA.127690.1 can target TraesCS2A02G518500.1 with a significant positive relationship of expression level (Fig. 7e), supporting that lncRNA.127690.1-TraesCS2A02G518500.1 pair may play a key role in regulating heading in wheat.

### LncRNA-mRNA pair associated with spike length

**Fig. 8:**
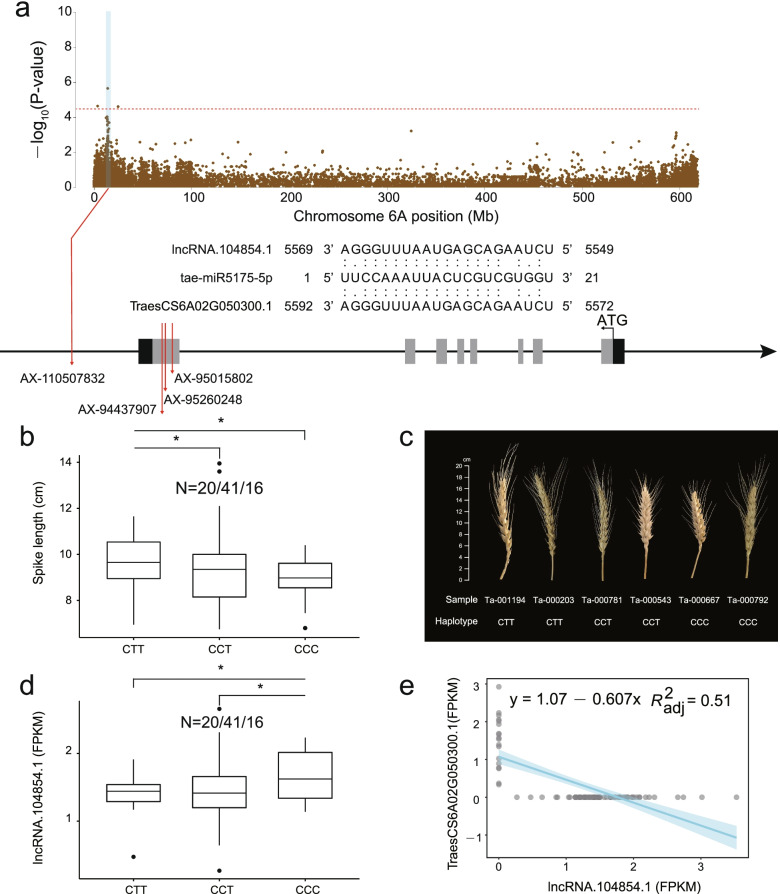
Key lncRNA-mRNA pair related to spike length. **a** Manhattan plot of spike length in chromosome 6A; **b** The phenotype value of spike length under each haplotype; **c** Agronomic performance of different haplotypes; **d** The expression level of lncRNA.104854.1 under each haplotype; **e** Regression analysis of the expression levels between lncRNA.104854.1 and TraesCS6A02G050300.1. (*, *P*-value ≤ 0.05)

A total of 43 lncRNAs and 33 target mRNAs were found to be related to spike length in wheat. A GWAS signal that associated with spike length (AX-110507832, Chr6A:24725411, *P*-value = 2.39E-05) was found in the downstream of lncRNA.104854.1 and its *cis-*regulatory mRNA (TraesCS6A02G050300.1) (Fig. 8a). Meanwhile, three non-synonymous SNP variations were found in the eighth exon region of TraesCS6A02G050300.1, resulting in three main haplotypes in this gene (Hap1: CTT, Hap2: CCT, Hap3: CCC) (Fig. 8a). Phenotypic investigation found that the spike length of Hap1 was the longest, followed by Hap2 and Hap3 (Fig. 8b and c). Interestingly, lncRNA.104854.1 can compete with tae-miR5175-5p to bind to TraesCS6A02G050300.1. The expression level of lncRNA.104854.1 in Hap1 was the lowest, followed by Hap2 and Hap3 (Fig. 8d). Notably, the expression level of lncRNA.104854.1 and TraesCS6A02G050300.1 showed the significantly negative relationship (Fig. 8e). TraesCS6A02G050300, encoding a autophagy protein, is an ortholog of *AtATG5.* Previous studies have demonstrated that autophagy protein played the crucial role in plant growth and development and *AtATG5* conjugated with *AtATG12* to form the ATG12-ATG5 module to control nutrient recycling in Arabidopsis [29, 30]. It suggested that lncRNA.104854.1-TraesCS6A02G050300.1 might be a key regulatory module to regulate spike length in wheat.

## Discussion

Although lncRNA has been widely reported to function as the important regulators in diverse biological processes in various plant species, including defense-response and development, a few studies were performed to link lncRNAs with yield-traits in wheat. Meanwhile, for the single genotype data, population analysis used multi-genotypes’ data to represent the more comprehensive and accurate landscape [31, 32]. Here, we systematically identified the lncRNA landscape in wheat spike through analyzing 186 RNA-seq data from 93 elite Chinese varieties and landraces. Based on two coding capability prediction software and PFAM database, the coding ability of all the assembled transcripts was preliminarily predicted. To confirm the accuracy of lncRNA and its expression in the population, we only remained the lncRNA with the expression level and non-coding potential. A total of 35,913 expressed lncRNA were identified, of which 11.5% were found in plant lncRNA database. Comparative analysis showed that lncRNA and protein-coding transcripts displayed the obvious difference in size, exon number, isoform number, GC content and expression level, which were consistent with previous studies [10, 22, 33]. Similarly, there were also some lincRNA within centromeric regions and the abundance of lincRNA was related to the length of centromeric regions, indicating the potential function of lincRNA in the reproductive development of wheat.

We further investigated the sequence conservation of lncRNAs between wheat and other eight plant species and higher conservation was found in the species with closer evolutionary relationship. Additionally, the genetic variations of these lncRNA were also analyzed based on the whole genome re-sequencing data of 261 *Triticum* and *Aegilops* samples [23]. The rapid loss of genetic diversity in lncRNAs was found in the process of wheat domestication, indicating severe bottlenecks during the process of wheat domestication and polyploidization. However, the genetic diversity of lncRNAs on B and D subgenomes displayed to increase from landrace improved into varieties, perhaps due to hybridization with its wild relatives during modern breeding processes. Overall, lncRNAs was subjected to genetic bottleneck events, which is similar to protein coding genes, in the process of wheat evolution.

Several introgression associating with significant agronomic traits have been identified in crops, such as rice, soybean, durum wheat and bread wheat [34–36]. In this study, according to the physical position of lncRNA, some lncRNAs were also co-located in introgression regions and QTL intervals. The 1BL/1RS translocation line is the most important and widely-used introgression material, which is formed by translocate rye 1RS chromosome arm to common wheat 1BL [37, 38]. 1BL/1RS line is considered to contribute significantly to wheat yield [39]. In our results, most lncRNAs and their targets were located on 1B chromosome, of which 65 loci were located in 1B/1R introgression interval and mainly associated with the heading and spikelet number per spike. Both 1B/1R and non-1B/1R translocation lines were contained in our samples. Thus, the 1B/1R-special lncRNA were identified and they displayed high expression in 1B/1R group but low or even no expression in non-1B/1R lines. Therefore, our results suggested that lncRNA on wheat 1RS arm was also translocated during 1R translocation (Additional file 2: Figure S5 and S6), and they played the role in regulating head stage and spikelet number of 1B/1R lines.

Autophagy protein played an important role in regulating plant growth and development as well as in stress response [40]. In Arabidopsis, *ATG5* can be conjugated with *ATG12*, and it played an important role in plant nutrient cycling pathways. Moreover, the chemically inducible complementation system in ATG5 knockout Arabidopsis demonstrated that accumulated superoxide requires degradation by autophagy, suggesting that autophagy is essential for the quality control of peroxisomes and for plant development under natural growth conditions [30]. In this study, TraesCS6A02G050300, which is the ortholog of *AtATG5*, was found to associate with spike length based on GWAS signals. The haplotype CTT has the longest spike length and higher expression of TraesCS6A02G050300, supporting the important function of this haplotype in regulating the spike length of wheat. Although *ATG5* has been reported to be involved in plant development, its function in wheat needs to be further verified, especially its effect on wheat spike-related traits. As a potential regulator of *ATG5* (TraesCS6A02G050300.1), lncRNA.104854.1 might use for genetic improvement and breeding together with *ATG5*, but the interaction between *ATG5* and lncRNA.104854.1 also needed to be further verified in wheat. Additionally, lncRNA.127690.1-TraesCS2A02G518500.1 module was found to regulate heading in wheat. TraesCS2A02G518500.1 is the ortholog of *OsPMEI*, which has been demonstrated to associate with heading in rice [28]. Further functional study of lncRNA.127690.1-TraesCS2A02G518500.1 module could contribute to better understand the molecular mechanism underlying heading in wheat from the perspective of lncRNA.

## Conclusions

To sum, we identified 35,913 lncRNAs as well as 1,619 lncRNA-mRNA pairs based on population RNA-seq dataset, presenting a comprehensive lncRNA landscape in wheat spike. We also found that these lncRNAs suffered a genetic bottleneck and displayed genetic introgression during wheat evolution. Moreover, 170 lncRNAs and 167 mRNA targets were identified to be associated with yield-related traits, including endogenous competing lncRNA. These findings provided an overall view on lncRNA for wheat genetic improvement from the transcriptional regulation level.

## Methods

### Plant materials and data collection

A total of 93 representative wheat genotypes were used in this study (Additional file 1: Table S13) [41], which were grown in the Nanyang (NY; 34° 38′ N, 112° 29′ E) and Luoyang (LY; 32° 54′ N, 112° 25′ E) trial, China in the 2018–2019 cropping season with the completely consistent weather and soil environment in the same trial. Each genotype was planted into one plot consisted of six rows with the length of 2 m and randomized block design was used with normal field management.

At the heading stage, three spikes from different individuals of each accession were collected and then bulked together for subsequent experiments and RNA sequencing. All samples were collected from 9 to 11 a.m. in the field. Total RNA of each sample was isolated using the Plant Tissue RNA Isolation kit following the instructor’s protocol (Qiagen, Germany). RNA-seq was performed using Illumina® HiSeq X Ten platform with paired-end 150 bp (Novogene, China). RNA-seq data has been deposited into Genome Sequence Archive (GSA) database with the accession number of PRJCA004969 (https://ngdc.cncb.ac.cn/bioproject/browse/PRJCA004969).

The spike-related traits, including spike length (SL), grain width (GW), grain number per spike (GN), spikelet number per spike (SNS) and heading date (HD) were investigated in the field in the 2018–2019 cropping season, Six individuals of each genotypes were randomly selected for agronomic trait evaluation.

The re-sequencing data of *Triticum* and *Aegilops* populations was downloaded from the Genome Variation Map published by Zhou et al*.* [23]. The introgression region and known QTLs region were obtained from the previous study of Cheng et al*.* [27]. The known QTLs included grain color (GC), grain yield (GY), plant height (PH), heading date (HD), kernel number per spike (KNS), kernel weight (KW), kernel length (KL), leaf spot disease (LSD), reaction to leaf rust (LR), normalized water index (NWI), spikelet number per spike (SNS), reaction to *Puccinia graminis* Pers (SR), sprouting, reaction to *Puccinia striiformis* Westend (YR) [27].

### RNA-seq analysis and lncRNAs identification

RNA-seq data was aligned to the wheat genome (IWGSC Refseq v1.1) using HISAT2 v2.2.1 [42] with default parameters. Novel and reference transcripts were assembled using StringTie v2.1.4 [43]. GTF file of each sample was merged by StringTie-merge tool and expression levels of transcripts were evaluated. These transcripts with normalized FPKM larger than 0.1 across at least 20% of the samples were considered as the expressed ones in this study. Transcripts length larger than 200 bp in length were retained for predicting coding ability using CPC and LGC with default parameters, respectively [21, 44, 45]. The PFAM database was further used to validate the protein coding ability of the transcripts. Then, Gffcompare was used to compare these transcripts with reference transcript. Finally, transcripts that belong to I (full contained within a reference intron), J (multi-exon with at least one junction match), O (other same strand overlap with reference exons), and U (unknown or intergenic) were considered as candidate lncRNA [4, 21]. All expressed lncRNA were retained for subsequent analysis.

### Identification of homologous lncRNAs and sequences conservation

LncRNA datasets of *A. thaliana* and other cereal crops (rice, barley, maize and sorghum) were retrieved from CANTATAdb 2.0 (http://cantata.amu.edu.pl/download.php) database and IC4R information (ftp://download.big.ac.cn/bigd/GEN/IC4R-2018/1-Separated-Data/1-RNA-SeqSupported/). Homologous sequences of between them were identified using BLASTn with E-value < 1E-05.

The wheat lncRNAs were aligned with other genomes using GMAP tool. The best hit of each lncRNA was extracted and that with conservation score > 0.6 [46] were used for subsequently analysis. The single copy lncRNA was used to constructed the maximum likelihood species tree using MEGA-X, with the parameter set to bootstrap of 1000 and Hasegawa-Kishino-Yano model and Gamma Distributed (G).

### Genetic diversity calculation and haplotype construction

Genetic variation datasets contained 261 *Triticum* and *Aegilops* accessions with the detailed information as follows: A1: wild einkorn wheat (*Triticum monococcum* L. ssp. *aegilopoides*), 31 accessions; A2: domesticated einkorn wheat (*Triticum monococcum* L. ssp. *monococcum*), 31 accessions; A3: urartu wheat (*Triticum urartu*), 29 accessions; AB1: wild emmer wheat (*Triticum turgidum* L. ssp. *dicoccoides*), 28 accessions; AB2: domesticated emmer wheat (*Triticum turgidum* L. ssp. *dicoccon*), 29 accessions; AB3: durum wheat (*Triticum turgidum* L. ssp. *durum*), 13 accessions; ABD1: landrace of bread wheat (*Triticum aestivum* L. ssp. *aestivum*), 45 accessions; ABD2: cultivar of bread wheat (*Triticum aestivum* L. ssp. *aestivum*), 25 accessions; D1: *Aegilops tauschii*, 30 accessions [23]. SNPs in lncRNA and mRNA were extracted based on the chromosome location using in-house Python script. Then, nucleotide diversity (π) and fixation index (*F*_st_) were calculated using VCFtools v0.1.16 with a sliding window of 50 Kb. The haplotype of lncRNAs and target mRNAs were constructed in nine subgroups using in-house Python scripts. Haplotypes with > 50% of the total number of subgroups were considered the major haplotype [47].

### Construction of miRNA/lncRNA-associated ceRNA networks

The target genes of lncRNA can be classified into *cis-* or *trans-* ones. The protein coding gene within 100 kb around lncRNA is considered as *cis-*regulatory gene [21, 48]. In contrary, the distance between *trans-*regulatory gene and lncRNA is more than 100 kb. All lncRNAs without ‘N’ were included in the analysis. Meanwhile, LncTar [49] was used to calculate the free energy of lncRNA-mRNA pairs. Whether cis or trans, the expression level between lncRNA and target gene must be significantly correlated (spearman correlation |*r*|> 0.9, *p* < 0.05). miRNA binding sites of lncRNA and mRNA were predicted using psRNATarget [50] with default parameters. The interrelationship of lncRNA, miRNA and mRNA were identified using in-house Python script. Transcript secondary structure was predicted using RNAfold tool.

### GWAS analysis of lncRNA-mRNA pairs

Wheat 660 K genotyping datasets of all accessions from previous study was used to perform the GWAS analysis [41]. The physical positions of the SNPs were obtained from the *Triticeae* Multi-omics Center website (http://202.194.139.32/). Based on the compressed MLM model [51], GWAS was conducted using TASSEL v5.0, and the *P*-value = 1/Ne (21,868, the effective SNP number of independent tests) was determined to be the threshold for a significant association [52, 53]. Then, according to the LD decay distance (Additional file 2: Figure S8), lncRNAs within 5 MB (r^2^ ≥ 0.2) upstream and downstream of trait-associated SNPs were considered as the potential candidate for the target traits.

### GO and KEGG enrichment analysis

The over-represented GO terms were determined using the online toolkit AgriGO v2.0 [54] with the false discovery rate (FDR) < 0.05. KOBAS v3.0 [55] was used for KEGG pathways enrichment with default parameters.

### Validation by qRT-PCR analysis

The qRT-PCR was performed for 10 randomly selected lncRNAs in five 1B/1R lines and five non-1B/1R lines. All reactions were performed with three independent technological replicates. RNA Easy Fast Plant Tissue Kit (Tiangen, China) was used to extract total RNA from twenty samples and RT Master Mix Perfect Real-Time kit (Takara, Japan) was used to synthesize cDNA according to the manufacturer’s instruction. qPCR reaction was performed on a QuantStudioTM 7 Flex System (Thermo Fisher Scientific, USA) using SYBR® Green Premix Pro Taq HS qPCR Kit (Accurate Biology, China) with the following thermal cycling conditions: 95 ℃ for 30 s followed by 40 cycles of 95 ℃ for 3 s, 60 ℃ for 30 s. The expression levels were calculated using the 2^−ΔΔCT^ method with *TaActin2* as the internal reference gene. The primers used in this study were listed in the supplementary file (Additional file 1: Table S14).

## Supplementary Information

**Additional file 1: Table S1.** Summary of all lncRNAs. **Table S2.** Summary of all lncRNA-mRNA pairs. **Table S3.** GO and KEGG enrichment of all target mRNAs. **Table S4.** Homologous lncRNAs between wheat and other species. **Table S5.** Sequence conservation of wheat lncRNAs based on results of whole-genome alignment. **Table S6.** Message of samples in population analysis. **Table S7.** Nucleotide diversity in each group. **Table S8.** Detail information of haplotype in lncRNA or target mRNA. (Yellow annotation indicates that gene has different haplotypes in different populations). **Table S9.** Interrelationship of ceRNA network between lncRNA, miRNA and mRNA. **Table S10.** Overlap between lncRNA or target mRNA and GWAS signals. **Table S11.** Overlap between lncRNA or target mRNA and known QTLs. **Table S12.** Overlap between lncRNA or target mRNA and introgression regions. **Table S13.** Information of 93 wheat lines. **Table S14.** Primers used for qRT-PCR analysis.

**Additional file 2:** **Figure S1.** Enrichment analysis of mRNA targets of lncRNAs. (a) GO enrichment analysis of target mRNAs. Different colors represent different GO term categories. (b) KEGG enrichment analysis of mRNA targets. Pathways were sorted by rich factor on the x-axis, which is determined by rich factor = (significant gene count of GO term)/(total gene count of GO term). **Figure S2.** Phylogenetic relationships of lncRNAs and target mRNAs on A subgenome. (a) Phylogenetic tree of lncRNA-mRNA pairs on A subgenome. (b) Phylogenetic tree of lncRNAs on A subgenome. (c). Phylogenetic tree of mRNAs on A subgenome. **Figure S3.** Phylogenetic relationships of lncRNAs and target mRNAs in B subgenome. (a) Phylogenetic tree of lncRNA-mRNA pairs on B subgenome. (b) Phylogenetic tree of lncRNAs on B subgenome. (c). Phylogenetic tree of mRNAs on B subgenome. **Figure S4.** Phylogenetic relationships of lncRNAs and target mRNAs in D subgenome. (a). Phylogenetic tree of lncRNA-mRNA pairs on D subgenome. (b) Phylogenetic tree of lncRNAs on D subgenome. (c). Phylogenetic tree of mRNAs on D subgenome. **Figure S5.** Expression and functional enrichment of lncRNAs and target mRNAs in chromosome 1BS. (a). Heatmap of expression levels of lncRNAs and target mRNAs in the 1B1R region. (b). Boxplots of expression levels of lncRNAs and target mRNAs in the 1B1R region in the two groups of samples. (c). GO and KEGG enrichment analysis of target mRNAs within 1B1R region. **Figure S6.** qRT-PCR validation of lncRNAs between 1B1R samples and non-1B1R samples. (a). qRT-PCR validation of 1B1R lineage-specific lncRNAs. (b). qRT-PCR validation of non-1B1R lineage-specific lncRNAs. The significance of expression level between 1B1R and non-1B1R groups was statistically analyzed by student's t-test. **Figure S7.** Phylogenetic relationships of TraesCS2A02G518500 in A subgenome. **Figure S8.** Genome-wide average LD decay estimated from 93 samples.

## Data Availability

The datasets supporting the conclusions of this article are included within the article and its additional files. RNA sequencing data used in this study has been deposited into Genome Sequence Archive (GSA) database with the accession number of PRJCA004969 (https://ngdc.cncb.ac.cn/bioproject/browse/PRJCA004969) and other data was provided in supporting information files.

## Abbreviations

LncRNAs: Long noncoding RNAs
qRT-PCR: Quantitative real-time polymerase chain reaction
ceRNA: Competing endogenous RNAs
TF: Transcription factor
GO: Gene ontology
KEGG: Kyoto encyclopedia of genes and genomes
