# Supplementary material for: Population transcriptomic analysis identifies the comprehensive lncRNAs landscape of spike in wheat (Triticum aestivum L.)
Source: BMC Plant Biol. 2022 Sep 21;22:450. doi: 10.1186/s12870-022-03828-x (PMC9490906; doi:10.1186/s12870-022-03828-x)
Supplement: Supplementary file 2 — Additional file 2: Figure S1. Enrichment analysis of mRNA targets of lncRNAs. (a) GO enrichment analysis of target mRNAs. Different colors represent different GO term categories. (b) KEGG enrichment analysis of mRNA targets. Pathways were sorted by rich factor on the x-axis, which is determined by rich factor = (significant gene count of GO term)/(total gene count of GO term). Figure S2. Phylogenetic relationships of lncRNAs and target mRNAs on A subgenome. (a) Phylogenetic tree of lncRNA-mRNA pairs on A subgenome. (b) Phylogenetic tree of lncRNAs on A subgenome. (c). Phylogenetic tree of mRNAs on A subgenome. Figure S3. Phylogenetic relationships of lncRNAs and target mRNAs in B subgenome. (a) Phylogenetic tree of lncRNA-mRNA pairs on B subgenome. (b) Phylogenetic tree of lncRNAs on B subgenome. (c). Phylogenetic tree of mRNAs on B subgenome. Figure S4. Phylogenetic relationships of lncRNAs and target mRNAs in D subgenome. (a). Phylogenetic tree of lncRNA-mRNA pairs on D subgenome. (b) Phylogenetic tree of lncRNAs on D subgenome. (c). Phylogenetic tree of mRNAs on D subgenome. Figure S5. Expression and functional enrichment of lncRNAs and target mRNAs in chromosome 1BS. (a). Heatmap of expression levels of lncRNAs and target mRNAs in the 1B1R region. (b). Boxplots of expression levels of lncRNAs and target mRNAs in the 1B1R region in the two groups of samples. (c). GO and KEGG enrichment analysis of target mRNAs within 1B1R region. Figure S6. qRT-PCR validation of lncRNAs between 1B1R samples and non-1B1R samples. (a). qRT-PCR validation of 1B1R lineage-specific lncRNAs. (b). qRT-PCR validation of non-1B1R lineage-specific lncRNAs. The significance of expression level between 1B1R and non-1B1R groups was statistically analyzed by student's t-test. Figure S7. Phylogenetic relationships of TraesCS2A02G518500 in A subgenome. Figure S8. Genome-wide average LD decay estimated from 93 samples. [file 12870_2022_3828_MOESM2_ESM.docx]

**
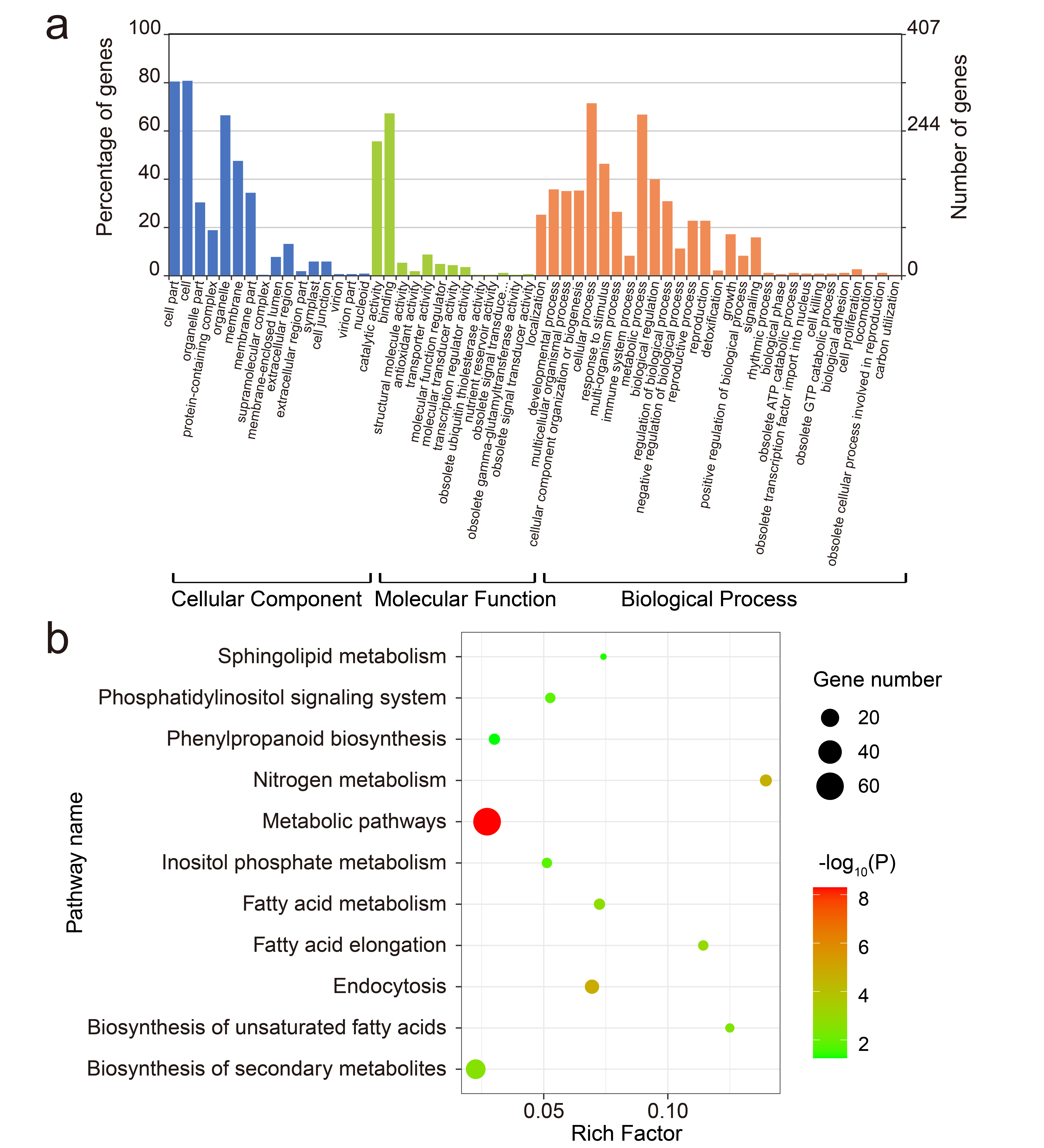
**

**Figure S1.** Enrichment analysis of mRNA targets of lncRNAs. (a) GO enrichment analysis of target mRNAs. Different colors represent different GO term categories. (b) KEGG enrichment analysis of mRNA targets. Pathways were sorted by rich factor on the x-axis, which is determined by rich factor = (significant gene count of GO term)/(total gene count of GO term).

**
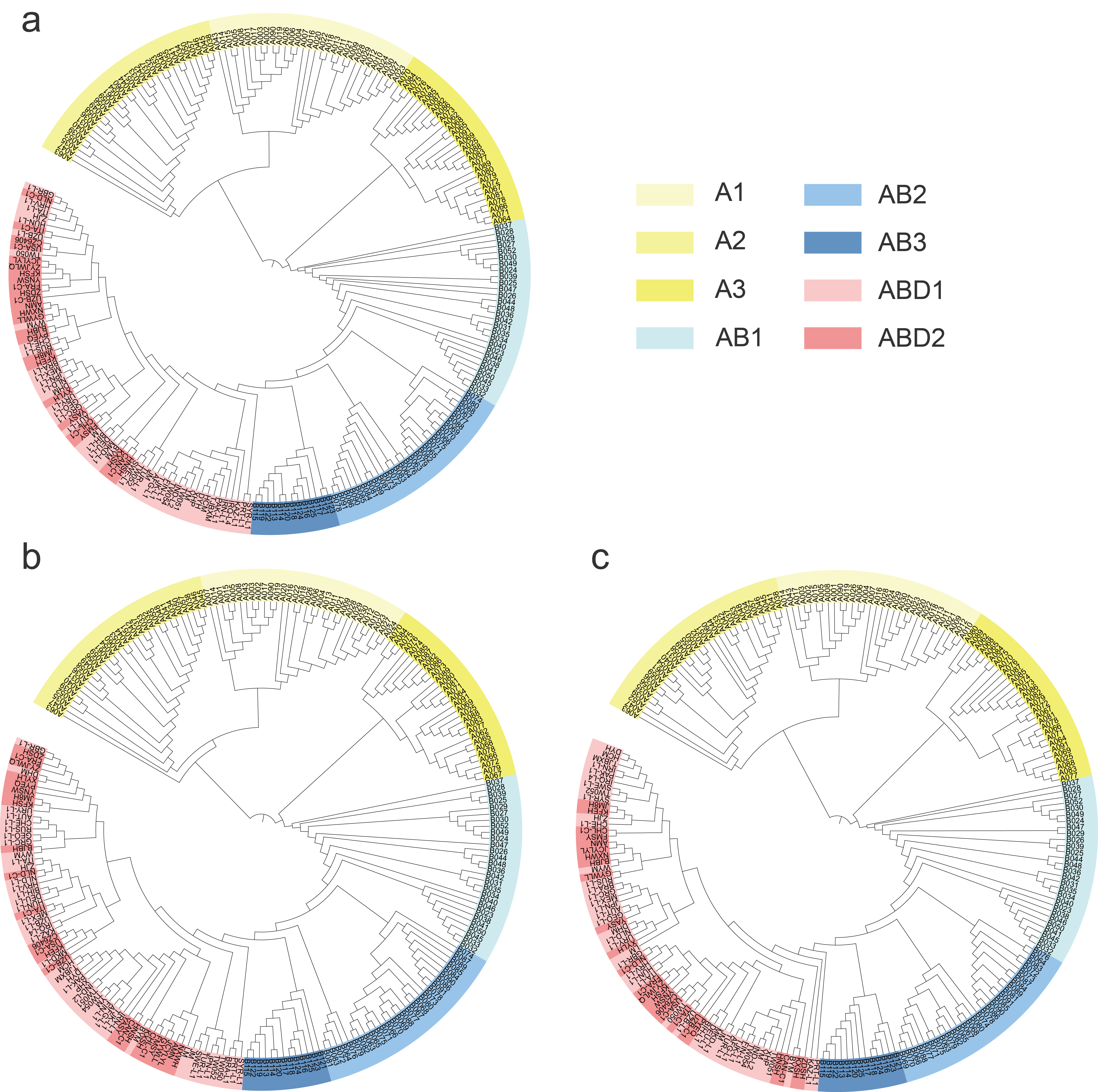
**

**Figure S2**. Phylogenetic relationships of lncRNAs and target mRNAs on A subgenome. (a) Phylogenetic tree of lncRNA-mRNA pairs on A subgenome. (b) Phylogenetic tree of lncRNAs on A subgenome. (c). Phylogenetic tree of mRNAs on A subgenome.

**
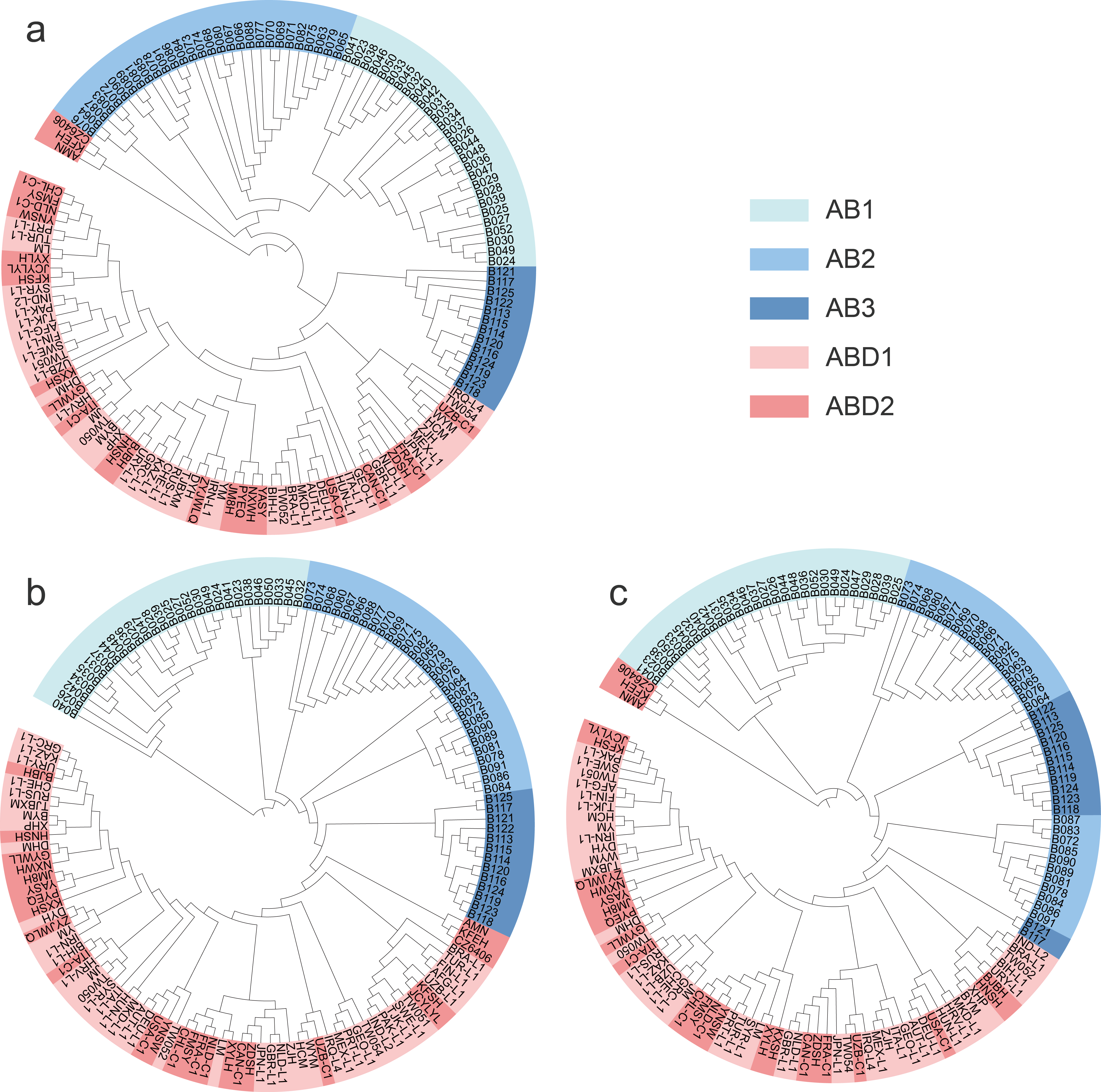
**

**Figure S3.** Phylogenetic relationships of lncRNAs and target mRNAs in B subgenome. (a) Phylogenetic tree of lncRNA-mRNA pairs on B subgenome. (b) Phylogenetic tree of lncRNAs on B subgenome. (c). Phylogenetic tree of mRNAs on B subgenome.

**
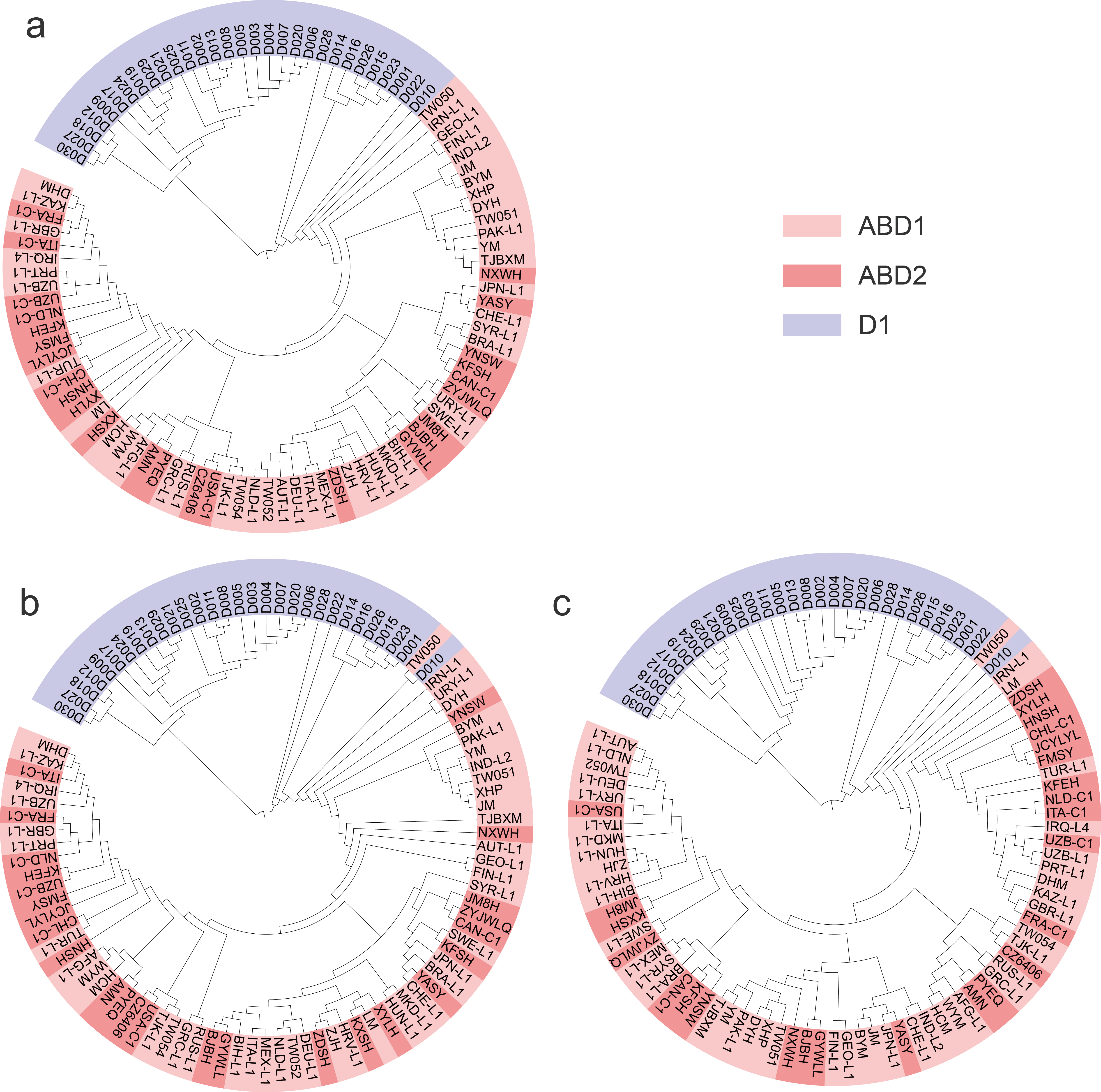
**

**Figure S4.** Phylogenetic relationships of lncRNAs and target mRNAs in D subgenome. (a). Phylogenetic tree of lncRNA-mRNA pairs on D subgenome. (b) Phylogenetic tree of lncRNAs on D subgenome. (c). Phylogenetic tree of mRNAs on D subgenome.

**
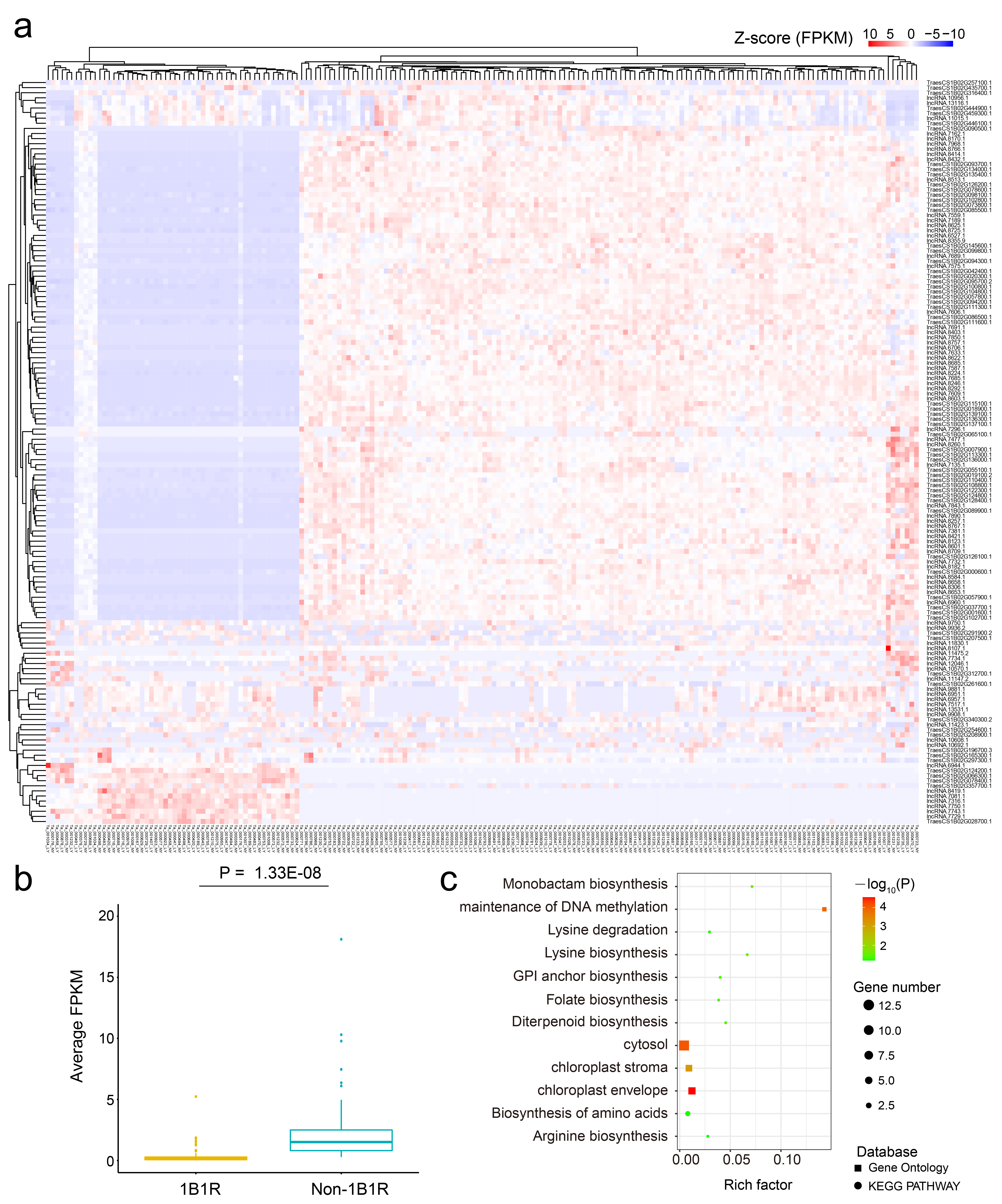
**

**Figure S5.** Expression and functional enrichment of lncRNAs and target mRNAs in chromosome 1BS. (a). Heatmap of expression levels of lncRNAs and target mRNAs in the 1B1R region. (b). Boxplots of expression levels of lncRNAs and target mRNAs in the 1B1R region in the two groups of samples. (c). GO and KEGG enrichment analysis of target mRNAs within 1B1R region. **
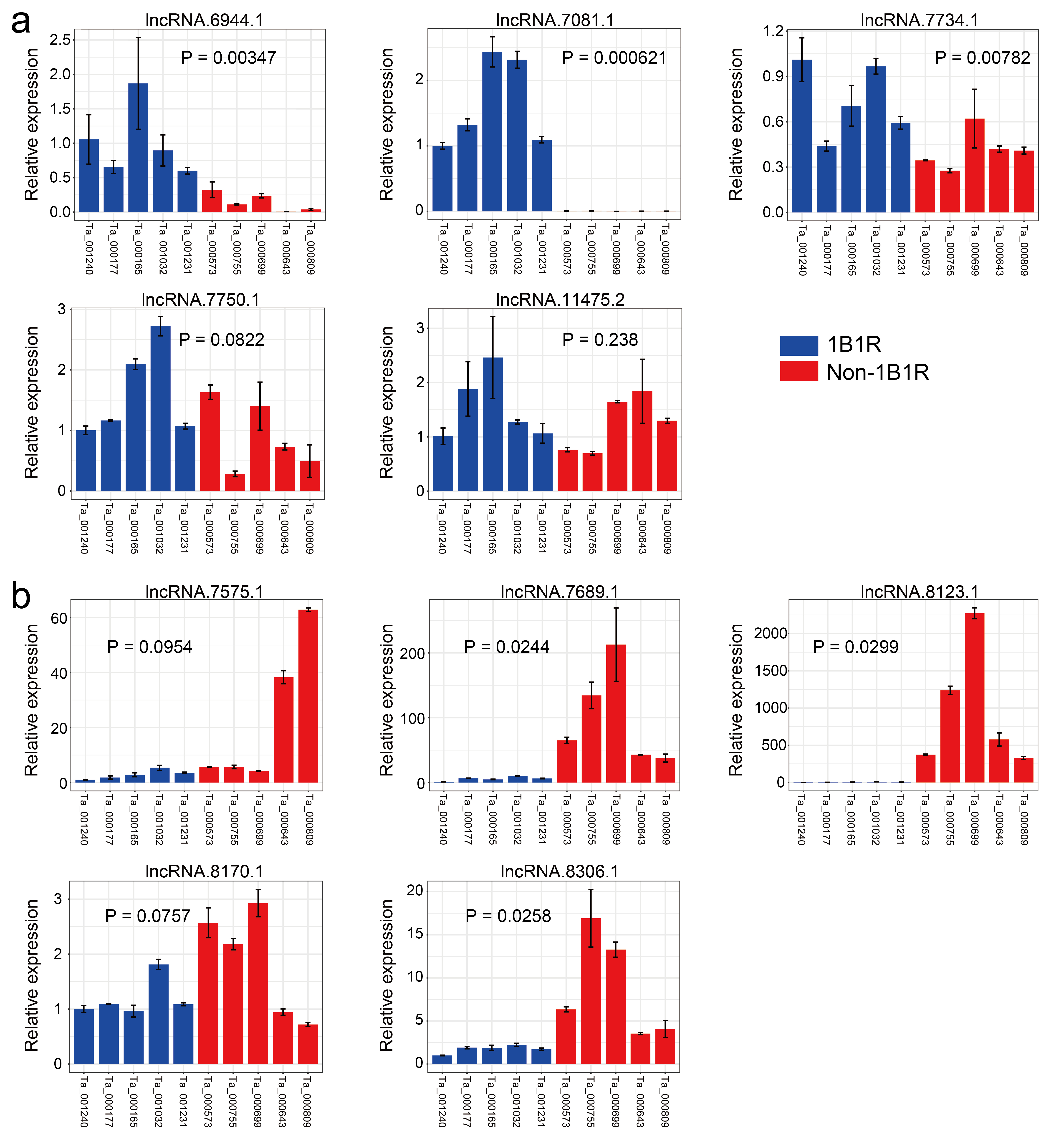
**

**Figure S6.** qRT-PCR validation of lncRNAs between 1B1R samples and non-1B1R samples. (a). qRT-PCR validation of 1B1R lineage-specific lncRNAs. (b). qRT-PCR validation of non-1B1R lineage-specific lncRNAs. The significance of expression level between 1B1R and non-1B1R groups was statistically analyzed by student's t-test.

**
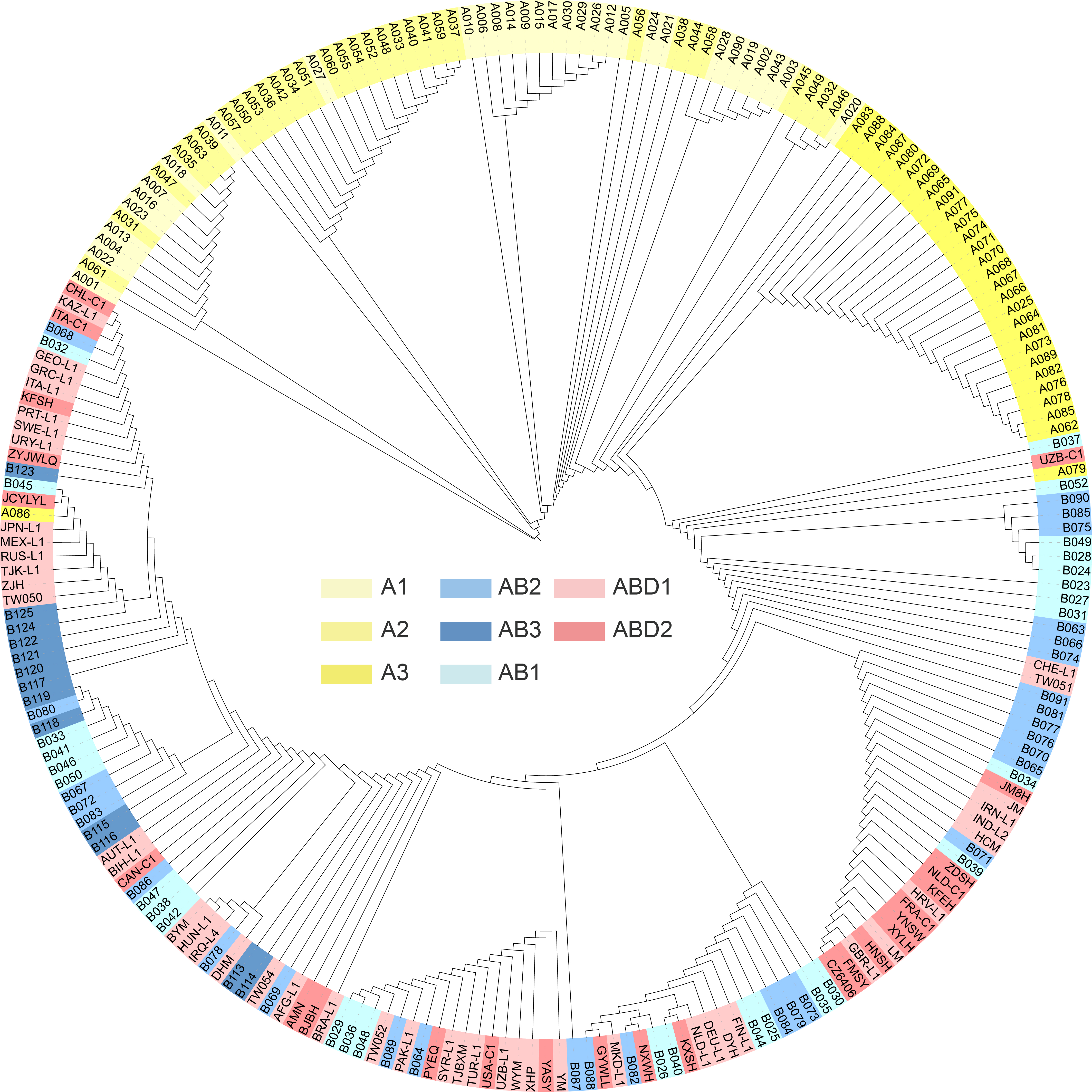
**

**Figure S7.** Phylogenetic relationships of TraesCS2A02G518500 in A subgenome.

**
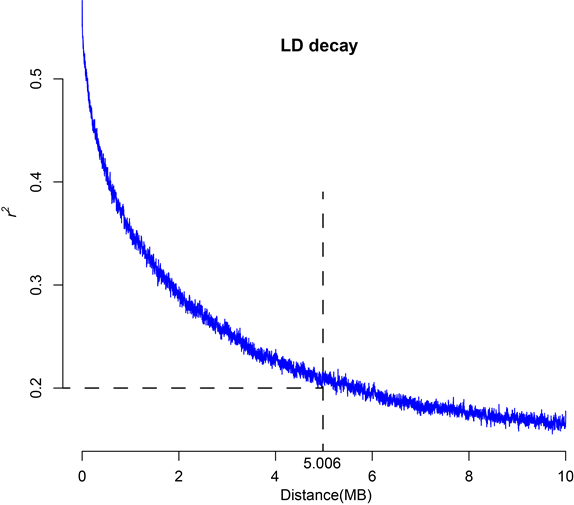
**

**Figure S8.** Genome-wide average LD decay estimated from 93 samples.
